# Comprehending Non-literal Language: Effects of Aging and Bilingualism

**DOI:** 10.3389/fpsyg.2018.02230

**Published:** 2018-11-22

**Authors:** Shamala Sundaray, Theodoros Marinis, Arpita Bose

**Affiliations:** ^1^School of Psychology and Clinical Language Sciences, University of Reading, Reading, United Kingdom; ^2^Department of Linguistics, University of Konstanz, Konstanz, Germany

**Keywords:** aging, bilingualism, executive control, metaphors, pragmatic inferences

## Abstract

A pressing issue that the twenty-first century is facing in many parts of the developed world is a rapidly aging population. Whilst several studies have looked at aging older adults and their language use in terms of vocabulary, syntax and sentence comprehension, few have focused on the comprehension of non-literal language (i.e., pragmatic inference-making) by aging older adults, and even fewer, if any, have explored the effects of bilingualism on pragmatic inferences of non-literal language by aging older bilinguals. Thus, the present study examined the effects of age(ing) and the effects of bilingualism on aging older adults' ability to infer non-literal meaning. Four groups of participants made up of monolingual English-speaking and bilingual English-Tamil speaking young (17–23 years) and older (60–83 years) adults were tested with pragmatic tasks that included non-conventional indirect requests, conversational implicatures, conventional metaphors and novel metaphors for both accuracy and efficiency in terms of response times. While the study did not find any significant difference between monolinguals and bilinguals on pragmatic inferences, there was a significant effect of age on one type of non-literal language tested: conventional metaphors. The effect of age was present only for the monolinguals with aging older monolinguals performing less well than the young monolinguals. Aging older bilingual adults were not affected by age whilst processing conventional metaphors. This suggests a bilingual advantage in pragmatic inferences of conventional metaphors.

## Introduction

Everyday communication involves not only literal language, but also the use of non-literal language, such as idioms, proverbs, metaphors, indirect requests, and conversational implicatures. To comprehend non-literal language, pragmatic inferences have to be made: the listener has to go beyond the literal meaning of the utterance and draw upon the situational context of the utterance as well as the listener's and speaker's knowledge of the world to arrive at the implied (non-literal) meaning. Pragmatic inferences are also thought to be cognitively more demanding because the listener has to both access their theory of mind to realize the speaker's communicative intentions (Champagne-Lavau and Joanette, 2009) and inhibit the literal meaning (Glucksberg et al., 2001) which becomes activated together with the implied meaning (Stewart and Heredia, 2002) during the processing of the non-literal language. Given that a great part of our daily conversations includes non-literal utterances, it is remarkable that listeners are able to comprehend them effortlessly and in great speed in spite of the high cognitive demands. This is true of healthy young adults who are in the peak of their cognitive abilities. However, it is unclear whether this is the case also for aging older adults, whose cognitive abilities are on the decline. Moreover, it is unclear whether the aging process affects the comprehension of non-literal language in monolingual and bilingual aging older adults in the same way given recent findings that show bilinguals having a cognitive reserve (Craik et al., 2010; Bialystok et al., 2013). The present paper fills these gaps by addressing how monolingual and bilingual healthy young and aging older adults comprehend non-literal language.

The general perception has been that the language abilities of aging older adults regress with each decade. However, research has revealed that regression is not in all language areas. Healthy aging older adults may face difficulty in understanding spoken discourse, experience problems retrieving words from the mental lexicon while speaking or increasingly suffer from tip-of-the-tongue state (Gollan and Brown, 2006; Thornton and Light, 2006; Burke and Shafto, 2008). On the other hand, they have been found to have a larger vocabulary size (Burke and Shafto, 2008; Bialystok and Luk, 2012; Kavé and Halamish, 2015), and to create more complex narratives than younger adults (Thornton and Light, 2006; Burke and Shafto, 2008). Healthy aging older adults have also been reported to use “high-level vocabulary and complex syntax” (Ulatowska et al., 1998, p. 628). In addition, sentence comprehension has been reported to be intact in old age (Tyler et al., 2009).

While much research has been aimed at aging older adults' understanding and production of vocabulary and grammatical structures at the sentential level and at times, discourse level (see Thornton and Light, 2006 for a comprehensive review), research into the pragmatic language abilities of aging older adults is comparatively rather scattered, if not impoverished. Thus, it is unclear whether or not aging older adults' pragmatic inferential abilities, which lead to correct meaning formation of non-literal languages, regresses much like some other aspects of the aging older adults' language.

Of the few studies that have investigated the comprehension of non-literal language by aging older adults, the focus has been on idioms (Westbury and Titone, 2011), proverbs (Nippold et al., 1997; Ulatowska et al., 1998; Uekermann et al., 2008) and metaphors (Newsome and Glucksberg, 2002; Qualls and Harris, 2003; Mashal et al., 2011). These studies, discussed below, have revealed contradictory or questionable findings in terms of the aging older adults' pragmatic inferential abilities.

A few of the aforementioned studies point to regression in aging older adults' pragmatic inferential abilities. Nippold et al. (1997) investigated the proverb comprehension abilities of 353 people aged between 13 and 79 years in Oregon using a Proverb Explanation Task. This task consisted of 24 proverbs which had received low familiarity ratings in Nippold and Haq (1996, cited in Nippold et al., 1997). The adolescents and adults read short stories with the proverbs appearing at the end and wrote down the meanings of the proverbs. While the study found proverb comprehension ability to decline in adults in their 60s (Nippold et al., 1997), the stories, based on one out of the two examples provided by the authors, required connective inferences. A failure to make the connective inference could potentially impede understanding of the proverbs under study. Uekermann et al. (2008) study of 105 healthy adults, 35 of whom were aging older adults between the ages of 60 and 79, led to a similar conclusion that aging older adults were impaired in proverb comprehension. The participants in this study had to, firstly, rate the familiarity of 32 German proverbs on a five-point Likert scale, and secondly, had to determine the non-literal meaning of these proverbs from four options which varied along “degree of abstraction” and “meaningfulness” (p. 35). On the other hand, other studies did not find any regression in aging older adults' non-literal language comprehension. Ulatowska et al. (1998), who had looked at 16 normally aging older monolingual speakers of American English in their 80s and 90s over a period of three years, found that there was no decline in proverb understanding and interpretation; instead there was an improvement for familiar proverbs and no significant changes for unfamiliar proverbs on the second testing after three years.

Metaphor comprehension too does not seem to regress with age. Aging older adults have been found to have access to metaphorical meaning (Morrone et al., 2010). Morrone et al. (2010) found their aging older participants aged 65 to 75 years making more errors and taking a longer time to reject the non-literal meaning of metaphors than the younger participants aged 21 to 30 years. This was believed to indicate that the aging older adults had access to the non-literal meanings of the metaphors. They posit that the non-literal meanings of the metaphors were likely activated and arrived at immediately, and thus needed to be inhibited; a decline in the inhibitory abilities of the aging older adults was deemed to lead to longer rejection times and more errors. Similarly, Newsome and Glucksberg (2002) found that the metaphor comprehension processes of aging older adults between the ages of 70 to 79 were not only seemingly intact, but also that the aging older adults were “as efficient as the younger adults (aged 17–21) in filtering out metaphor-irrelevant information” (p. 262). Newsome and Glucksberg presented the non-reversible metaphors and literal phrases in sentences as primes which were followed by metaphor-relevant and metaphor-irrelevant sentence probes with the last word of each prime beginning each sentence probe; participants had to judge whether the sentences made sense. Both young adults and aging older adults were better able to appreciate metaphor-relevant material after being primed by the metaphors and metaphor-irrelevant materials after being primed by the literal sentence primes.

In some instances, older adults have been found to possess superior pragmatic inferential abilities to young adults. Qualls and Harris (2003) investigated both younger (17–31 years) and older (54–73 years) African American adults' comprehension of non-literal language. This study revealed that the older adults have better comprehension of idioms and metonyms than the younger adults. However, Qualls and Harris (2003) had a number of important confounds in their study: the answer options for metonyms included metaphors, which themselves require pragmatic inferring. In addition, the metaphor items included both conventional and novel metaphors, both under the umbrella term of metaphors. This is problematic because processing of conventional and novel metaphors employ different cognitive mechanisms and appreciation of novel metaphors has been shown to be affected by age (Mashal et al., 2011). Lastly, the authors had included adults who were between 50 and 59 in their group of older adults. Whilst this definition of older adults is applicable to most African countries (World Health Organisation, 2002), it should not apply to African Americans who experience a longer life expectancy than and differ socially from the people in Africa; adults between 50 and 59 years of age would have better cognitive abilities than older adults, thus confounding the results.

Another important study on metaphors and aging older adults is the study by Mashal et al. (2011). Mashal et al. (2011) compared young and aging older adults in their appreciation of conventional and novel metaphoric expressions. Their first experiment, which was aimed at rating the plausibility of metaphors and literal expressions, revealed that the young adults regarded more metaphoric expressions as plausible than the aging older adults, with both groups not showing any significant difference for the plausibility rating of the literal and unrelated expressions. However, it is unclear whether the aging older adults found more of the novel metaphoric expressions as less (or more) plausible than the conventional ones; this they address in their second experiment that used different groups of young and aging older adults to examine if there was any age effect in terms of appreciating conventional versus novel metaphors. In this second experiment, the young and aging older adults had to rate the familiarity level of the 79 metaphoric expressions that were appreciated as plausible in the first experiment. Interestingly, the aging older adults rated more of the metaphoric expressions as being more familiar, appreciating them as being conventional. This was unlike the young adults who regarded the metaphoric expressions as being more novel. Expressions that were deemed as being highly novel by the young adults, were rated as being highly meaningless by the aging older adults. The study by Mashal et al. (2011) alludes to novel metaphor processing, unlike conventional metaphor processing, to be problematic in aging older adults.

The aforementioned studies, besides highlighting the contradictory findings with regard to aging older adults' non-literal language comprehension, also point to the possibility that different pragmatic inference-making strategies are employed depending upon the type of non-literal language encountered (Garcia, 2004). In addition, these studies either did not present the non-literal utterances within a situational context or presented them in texts that require connective inferences to be made. In our everyday social interactions, literal and non-literal utterances do not occur in isolation. These utterances are produced within specific contexts, and we unpack the meaning of these utterances based on these contexts. Thus, the failure to comprehend non-literal language in some of the studies looked at earlier could be due to the lack of context. To address these shortcomings, the present study focused on the comprehension of a range of non-literal language in the same groups of participants and included a situational context for each target utterance to increase the ecological validity of the task.

All the studies mentioned above have focused on monolingual aging older adults. Although an estimated 50% or more of the world's population is either bilingual or multilingual (Grosjean, 2010), there is a lack of studies investigating bilingual aging older adults' comprehension of non-literal language. Given the current debate about whether or not bilinguals have better cognitive abilities than monolinguals and, as established earlier, the cognitive demands of pragmatic inferring during non-literal language comprehension, it is important to investigate the comprehension of non-literal language by bilingual aging older adults. In the present study, ‘bilinguals' are defined based on Grosjean (2010), according to whom bilinguals are people “who use two or more languages (or dialects) in their everyday lives.” (p. 4).

A number of studies have found that bilinguals have better cognitive abilities than monolinguals in terms of better executive control functions across the lifespan (Bialystok et al., 2006; Bialystok and Craik, 2010; Luk et al., 2011) and working memory (Bialystok et al., 2004). Moreover, aging adults who might otherwise succumb to dementia or neurodegenerative disease(s) earlier are now being diagnosed later due to their bilingualism (Craik et al., 2010). This has led to the hypothesis that the accrued neurocognitive differences arising from bilingual language processing over the lifespan lead to neuroplastic changes in the bilingual brain which attenuate age-related cognitive decline (Bak et al., 2014; Baum and Titone, 2014, p. 859). In addition, studies have also found that the frontal and temporal lobes, where language functions take place, are of greater volume in bilinguals than monolinguals (Olsen et al., 2015).

However, several other studies were not able to find a bilingual cognitive advantage (Paap and Greenberg, 2013; Zahodne et al., 2014; Bogulski et al., 2015). For example, in contrast to researchers who found bilinguals to be in possession of superior inhibitory abilities, Kousaie and Phillips (2012), using the Color Stroop task, did not find a bilingual advantage for inhibitory control for either their young bilinguals or their old bilinguals in comparison to their monolingual counterparts. Likewise, Colzato et al. (2008) did not find any difference between the young monolinguals and young bilinguals in the Stop Signal inhibition task, although they did find the bilinguals to be better able to maintain action goals and use them to differentiate goal-related information leading to “more pronounced reactive inhibition of irrelevant information” (p. 302). Similarly, de Bruin et al. (2015), who had controlled for a number of variables such as education, socioeconomic status, intelligence, age of acquisition and immigration status, did not find a bilingual cognitive advantage for inhibitory control in their aging older adults regardless of whether they were active or inactive bilinguals. Yet other studies have found the age of acquisition of the second language to influence the bilingual cognitive advantage; Vega-Mendoza et al. (2015) found late acquisition of second language having a positive effect on inhibition. Given that the comprehension of non-literal language is cognitively more demanding, examining monolingual and bilingual aging older adults' comprehension of non-literal language can shed light on the debate surrounding the cognitive advantage in bilinguals.

The present study addresses the issues highlighted earlier by investigating the comprehension of non-literal utterances by monolingual and bilingual young and aging older adults. It aims to answer two research questions: (1) Is there an age effect on pragmatic inference-making? and (2) Is there a bilingual advantage in pragmatic inference-making?

This study focuses on three types of frequently occurring non-literal language: non-conventional indirect requests, conversational implicatures, and metaphors which are further divided into conventional and novel metaphors. The inclusion of different types of non-literal language will allow for greater insight to the pragmatic inferential abilities of healthy aging older adults. It is predicted that aging older adults will have pragmatic inferential abilities on par with young adults for some, but not all, non-literal language types.

Given that a number of studies have argued that L1 and L2 proficiency, age of L2 acquisition, language dominance, and L1 or L2 dominant linguistic environment that the bilinguals live in ought to be taken into account when studying bilinguals (van Hell and Poarch, 2014; Dong and Li, 2015; Mishra, 2015; Titone et al., 2015), the present study controls for age of acquisition, vocabulary knowledge, verbal fluency (see Perani et al., 2003), education, socioeconomic status, inhibition, intelligence, and processing speed, which is known to slow down with age (Salthouse, 1996) as well as verbal short-term memory and working memory, which are believed to play vital roles in discourse processing and comprehension (Hasher and Zacks, 1988).

## Materials and methods

### Participants

Seventy-three healthy adults participated in this study: 19 monolingual English-speaking young adults (mean age = 19.47, *SD* = 0.7) and 20 monolingual English-speaking aging older adults (mean age = 69.9, *SD* = 6.8) from the United Kingdom as well as 19 bilingual English-Tamil-speaking young adults (mean age = 21.02, *SD* = 1.58) and 15 bilingual English-Tamil-speaking aging older adults (mean age = 67.01, *SD* = 4.39) from Singapore. Table 1 shows the demographic information of all four groups. All aging older adults were screened with the Mini Mental State Examination (MMSE) to rule out the onset of dementia or mild cognitive impairment; the cut-off of 27 was used based on a study conducted by O'Bryant et al. (2008) on the sensitivity of the MMSE. Table 1 shows the groups' mean scores on the MMSE. None of the aging older adults had a score of <27 on the MMSE.

**Table 1:** Demographic statistics of all participants.

**Demographic characteristics**		**Monolinguals**		**Bilinguals**	
		**Young** **(*n* = 19)**	**Old** **(*n* = 20)**	**Young^#^** **(*n* = 18)**	**Old** **(*n* = 15)**
Gender (M, F)		3, 16	10, 10	7, 11 [7, 12]	6, 9
Age	Mean *(SD)*	19.47 (0.7)	69.9 (6.8)	20.93 (1.57) [21.02 (1.58)]	67.01 (4.39)
	Min-Max	18–21	60–83	17–23	60–78
Education	Mean *(SD)*	14.97 (0.63)	14.4 (3.58)	15.83 (1.54) [15.89 (1.52)]	13.3 (3.63)
	Min-Max	14–16	10–20	14–19	7–18
MMSE	Mean *(SD)*	NA	28.8 (1.24)	NA	28.67 (1.05)
	Min-Max	NA	27–30	NA	27–30
CIMS	Mean *(SD)*	NA	11.65 (0.67)	NA	11.33 (0.98)
	Min-Max		10–12		9–12

*MMSE, Mini Mental State Examination; CIMS, Complex Ideational Materials Subtest; YM, Young monolinguals; YB, Young bilinguals; OM, Old monolinguals; OB, Old bilinguals*.

# *One bilingual young adult was excluded from the final analysis of the English pragmatic task because of equipment failure during this task. [ ] indicates data for n = 19 for young bilinguals*.

All participants completed the Language History and Use Questionnaire (LHUQ), an adaptation of the Language History Questionnaire of the Brain, Language, and Computation Lab, Penn State University (Li et al., 2006). The LHUQ consisted of 22 items which gather information such as the age of language acquisition, self-assessed language proficiency, and L1 and L2 frequency of use and code switching among other questions that elicit the participants' age, sex and socioeconomic status (SES) (years of formal education as an indication of SES). Table 2 provides the results of the LHUQ pertaining to age of language acquisition and language usage.

**Table 2:** Linguistic characteristics of participants derived from the LHUQ according to groups.

**Linguistic characteristics**		**YM** **(*N* = 19)**	**OM** **(*N* = 20)**	**YB** **(*N* = 19)**	**OB** **(*N* = 15)**
Age of Acquisition of English (in years)	0–5	19	20	17	2
	6–10	0	0	2	10
	11–19	0	0	0	3
Age of Acquisition of Tamil or other language (in years)	0–5	0	0	18	15
	6–10 11–19 20>	0 2 0	0 5 3	1 0 0	0 0 0
Conversing in English^∧^(hours/day)	Mean *(SD)*	13.95 (4.2)	10.73 (3.45)	10.08 (4.19)	5.2 (3.9)
	Min-Max	2.5–16	1.5–14	3–17	0.3–12
Conversing in Tamil or other language (hours/day)	Mean *(SD)*	0.5 (0.0)	0 (0)	4.4 (3.52)	6.12 (5.48)
	Min-Max	0.5–0.5	0	0–11	0.3–16

∧ *Monolingual young and older participants, who chose to state “English only” or “English All Day” when asked on the LHUQ to state the number of hours (out of 24 h per day) that they communicate with various groups of people in the languages they know, were assigned 16 and 12 h, respectively to match the total hours stated by their age cohorts*.

All monolingual participants were native speakers of British English. Some of the monolingual participants indicated on the LHUQ that they were aware of one or more foreign languages; these were learnt in a classroom setting around the age of 11 and later at school or after the age of 19 for work. Only two young monolinguals reported using their additional language. The use was only for half an hour out of 24 per day and not on a daily basis and therefore they were included in the monolingual group based on Grosjean's (2010) definition of bilinguals. All bilingual participants were speakers of Standard Singapore English and Standard Spoken Tamil; both English and Tamil were used in the homes of all bilingual participants. All, but four, of the young bilinguals reported that English was acquired from birth; two of the young bilinguals acquired English at the age of five, while the other two began acquiring English once in school at ages six and seven when they started school. Most of the older bilinguals began acquiring English from around the age of six, except for three older bilinguals who began learning English at the age of 12 in a formal school setting before migrating to Singapore as young adults. Given that English is widely used in public life in Singapore, all learners were exposed to English in a naturalistic environment, including these three older bilinguals. To address the potential role of age of acquisition acting as a confounding factor, it was included as a covariate in the analyses of the pragmatic tasks.

The Complex Ideational Materials Subtest (CIMS) of the Boston Diagnostic Aphasia Evaluation (BDAE) (short version) was used to test participants' auditory English sentence comprehension. The task includes a total of six pairs of yes-no questions. Each question answered correctly was awarded 1 point giving rise to a total possible score of 12. Only the aging older adults were tested in the CIMS because of the significant difference between the aging older monolinguals' and bilinguals' age of acquisition of English.

The monolingual young adults were undergraduates from the Department of Psychology, University of Reading, and received course credits for their participation. The monolingual aging older adults were recruited via the University of Reading's Aging Research Panel and were reimbursed £10 for their transport. The bilingual young adults were recruited from the National University of Singapore, the Nanyang Technological University and Ngee Ann Polytechnic in Singapore. The bilingual aging older adults were recruited through visits at temples in Singapore and through personal contacts and were given gifts of fruits and biscuits for cultural reasons.

### Materials

#### Background tests

To be able to control for potential confounding factors resulting from differences between the groups on verbal and non-verbal abilities as well as processing speed, a large battery of background tests was carefully selected to record the participants' lexical and semantic knowledge, and cognitive abilities, including fluid intelligence, verbal short-term memory and working memory as well as processing speed. In terms of verbal abilities, the battery focused on lexical and semantic rather than grammatical abilities because the experimental pragmatic tasks relied heavily on lexical and semantic information and did not have any grammatical manipulations. Of course, grammatical abilities are relevant for all tasks involving the sentence and discourse level, but the battery was already very long.

##### Lexical and semantic measures

The Raven's Short Vocabulary Scale (RVS), consisting of 17 words increasing in difficulty in an ascending order, was used to measure lexical knowledge. All participants had to give the meanings of the words on the list; their answers were audio recorded, and later scored with a 0 if outright wrong, 1 if partially correct and 2 when totally correct. Because vocabulary acquisition is positively related to SES (Hoff, 2003; Fernald et al., 2013), the RVS was used as a covariate together with education to control for the SES of the participants.

A Tamil vocabulary list (TVL) was created with the help of a native Singapore Tamil speaker. The TVL, like the RVS, had 17 vocabulary words and increased in its level of difficulty as the bilingual participants progressed down the list. The TVL was scored in a similar manner to the RVS.

The English Verbal Fluency (EVF) test comprised of the English Letter Fluency (ELF) task and the English Semantic Category Fluency (ESCF) task. The ELF task measures vocabulary retrieval, and together with the ESCF task, also detects neuropsychological impairments and frontal disorders (Gladsjo et al., 1999). In the ELF task, all participants were instructed to provide as many words as possible that began with the letters F, A, and S in one minute each. They were also instructed to exclude proper nouns, such as names of people and places. In the ESCF task, the participants were instructed to state as many animals as they could in one minute; they were specifically instructed to leave out breeds of the same animal (e.g., Alsatian, German Shepard, and Pomeranian all being breeds of the animal “dog”).

The Tamil Verbal Fluency (TVF) test comprised of a Tamil Letter Fluency (TLF) task and a Tamil Semantic Category Fluency (TSCF) task. In the Tamil LF task, the bilingual participants were given the Tamil letters 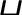 [p∧], 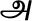 [∧], and 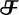 [s∧] and were similarly instructed as the English LF task, to provide as many words as possible that began with these letters in one minute each. They were also instructed to exclude proper nouns, such as names of people and places, and were provided with additional instructions where they were allowed to substitute the vowel sound [∧] in the syllabic consonants, 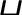 [p∧] and 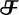 [s∧], with any of the other 11 vowels found in the Tamil alphabet.

The bilingual participants were required to complete both the EVF and the TVF. However, owing to the fact that Tamil speakers in Singapore seldom distinguish most animals by their breeds whilst speaking in Tamil, they were not instructed in the Tamil SCF to refrain from naming animals of the same breed.

##### Measures of cognitive abilities

The Stroop Arrow task (Blumenfeld and Marian, 2011) was used to measure participants' inhibitory abilities. The Stroop Arrow task has two stimulus dimensions: arrow direction and arrow location. These are either congruent, with right-facing arrow (or left-facing arrow) appearing on the right (or left) of the screen, or incongruent, with right-facing arrow (or left-facing arrow) appearing on the left (or right) of the screen. Participants had to respond to the direction of the arrow and ignore the location. For instance, for a right-facing arrow on the left screen, participants had to inhibit the reflex to press the key on the left for two accounts, one being the location of the arrow on screen and the other being the direction of the arrow. The Stroop Arrow task consisted of 40 congruent trials and 40 incongruent trials which were preceded by 12 practice trials. Each trial began with a black fixation cross which remained on the white screen for 800 ms and was followed by a blank white screen for 250 ms, before the stimulus appeared either on the left or the right of the white screen. The stimulus remained on screen for 1,000 ms or until a response key was hit. The trial ended with a blank screen that lasted for 500 ms, before a new trial began. The response keys were a “left-facing arrow” and a “right-facing arrow” which were overlaid on the “A” and “L” keys of a standard US keyboard, respectively. The Stroop Effect was obtained by subtracting the congruent reaction time from the incongruent reaction time for correct trials; a smaller Stroop Effect implies greater inhibitory control.

The Wechsler Adult Intelligence Scale (WAIS-III) Block Design was used to measure fluid intelligence and to control for between group differences on non-verbal IQ (de Oliveira et al., 2014). The WAIS-III Block Design required the participants to physically manipulate blocks to resemble the image shown to them. There was a total of nine images to reproduce using the blocks with five images being a two-by-two with a maximum time limit of 60 s and the remaining being a three-by-three with a maximum time limit of 120 s. Participants were scored according to the scoring system found in the WAIS-III Block Design where scores range between 4 and 7 for reproducing each image correctly within the time limit; for each image, the score obtained was inversely proportional to the time taken.

The forward and backward Digit Span (DS) tasks from the Wechsler Memory Scale (Revised) were used to test verbal short-term memory and working memory (Woods et al., 2011) because according to Hasher and Zacks (1988) they play vital role in discourse processing. In the forward digit span, participants were required to recall the digits in the order they were presented. In the backward digit span, participants were required to recall the sequence in the reverse order. Participants were given a score of one for each correct set of numbers recalled with a possible total score of 24.

The Number Comparison (NC) task (Salthouse and Babcock, 1991) was used to measure processing speed because the pragmatic task involved testing the response time. Participants had to decide if pairs of numbers were the same or different. There were 3 sets of 12 pairs of three, six and nine digits making a total of 36 items. All participants were timed separately for each set of pairs beginning with the three-digit pairs followed by the six-digit pairs and then the nine-digit pairs. Processing speed was calculated by first dividing the time taken to complete each set by the total number of items in the set (i.e., 12), and then multiplying that by the number of items that were correctly identified as being either same or different. The total number of correct items for the entire task was then divided by the total time taken for correct identification to give the processing speed (number of correct items per second).

#### Experimental pragmatic tasks

Two pragmatic tasks were created to measure a range of non-literal language and literal language: an English (EPrag) and a Tamil (TPrag) task. Each task was made up of five sets of 10 short stories to cover non-conventional indirect requests, conversational implicatures, conventional metaphors, novel metaphors, and literal utterances. Standard Singapore English is based on Standard British English; while there is no variation in the grammar, lexical differences do exist (Gupta, 2010, 2012; Leimgruber, 2011). Vocabulary that may have different meanings in the two varieties of English were avoided in the stories. Similarly, all stories were created to be culturally neutral, that is, the situational contexts were applicable to both Singapore and the United Kingdom. The English conventional metaphors were selected from a familiarity rating list administered to nine healthy aging monolingual English speakers aged 60 years and above in the United Kingdom and six healthy aging bilingual English-Tamil speakers aged 60 years and above in Singapore. Similarly, the Tamil conventional metaphors were selected from a familiarity rating list administered to the same group of aging bilingual English-Tamil speakers. Participants completed three practice trials before starting on the actual task.

Each trial consisted of a short dialog by or between a male and a female character that were accompanied by a line drawing to create a story. Participants heard the target utterances at the end of these short dialogs. Each story started with the narrator providing the setting (e.g., “At a party”) and background (e.g., “Jill is at a party.”) and ended with a multiple-choice comprehension question in the format of “What will < story character's name or gender> say or do next?”. Participants heard the narrator reading out the questions and the four options as well as seeing the questions and options displayed on the screen below the line drawings. The questions and options for EPrag were typed onto the slide as text, whereas the questions and their answer options for Tamil had to be handwritten and uploaded as images because the experiment software did not support the Tamil script font. The complete story board for the EPrag task can be found in the Supplementary Material.

Each option can be categorized under one of four types: (a) inferred meaning, (b) literal meaning, (c) possible, but wrong reaction and (d) wrong answer. There were two “wrong answers” for the literal category as there are no inferred meanings for the literal target utterances. Participants pressed the corresponding key on the keyboard to record their answers, after which a new slide with the words “Next story?” appeared on the screen. Pressing the space bar then brought the participants to the next slide which had a fixation cross for 250 ms before a new story begun.

The dependent variables—accuracy scores and time taken to respond (TTR) (in seconds)—were recorded for each of the non-literal language types (i.e., non-conventional indirect requests, conversational implicatures, conventional metaphors, and novel metaphors) and literal utterances. The TTR measure was calculated only for correct responses for each non-literal and literal language type tested.

### Procedure

The Pragmatic tasks were run using E-prime 2.0 Professional on an Acer Aspire 4820T laptop with an Intel® CoreTM i5 processor 4.30 M and a 14.0-inch HD LED LCD screen. Participants were tested individually in separate sessions. The bilingual participants completed the English and Tamil tasks in separate sessions. The bilinguals' testing sessions were counterbalanced by language; the English and Tamil sessions were spaced apart by two to three weeks.

### Data analyses

The study has set out to answer two research questions: (1) “Is there an age effect on pragmatic inference-making?,” and (2) “Is there a bilingual advantage in pragmatic inference-making?.” Language Group (monolingual, bilingual) and Age (young, old) were the independent variables for this study.

The age of acquisition of English and Tamil and CIMS scores were analyzed with a Mann-Whitney test. Age, education and the variables arising from the background tests were analyzed with a two-way univariate analysis of variance (ANOVA) with Age and Language Group as factors. The MMSE was analyzed with a one-way ANOVA with Language Group as the independent variable. Variables arising from the Tamil background tests were analyzed with a one-way ANOVA with Age as the independent variable.

Each of the pragmatic tasks (the EPrag and TPrag tasks) had five dependent variables for the accuracy and five for the TTRs, corresponding to the five pragmatic conditions (non-conventional indirect requests, conversational implicatures, conventional metaphors, novel metaphors and literal utterances).

For the EPrag task, a two-way multivariate analysis of covariance (MANCOVA) was used to test the effects of Age and Language Group on the EPrag accuracy scores (i.e., arising from the non-conventional indirect requests, conversational implicatures, conventional metaphors, novel metaphors and literal utterances) whilst controlling for potential effects of socioeconomic status, verbal IQ, education, inhibition, verbal short-term memory and working memory as well as age of acquisition of English that may affect the participants' inferential abilities. A similar analysis was conducted on the EPrag TTRs with Number Comparison as an additional covariate to control for the differing processing speed of the groups. Planned pairwise comparisons were conducted to compare differences between young and aging older adults, and monolinguals and bilinguals for each pragmatic condition separately.

For the TPrag task, a one-way MANCOVA was run to test for effects of Age on the TPrag accuracy scores (arising from the non-conventional indirect requests, conversational implicatures, conventional metaphors, novel metaphors, and literal utterances) with Education, Tamil Vocabulary List, Stroop Arrow, Block Design, Tamil Verbal Fluency, Age of Acquisition of Tamil and Digit Span as covariates. The covariates were included to control for socioeconomic status, verbal IQ, differing educational levels between groups, inhibition, verbal short-term memory, and working memory that can potentially affect inferential abilities, and to reduce error variances. Similarly, a one-way MANCOVA was conducted on the TPrag TTRs with Number Comparison as an additional covariate to control for differing processing speed of the groups. Finally, planned pairwise comparisons were conducted to compare differences between young and aging older bilingual adults for each pragmatic condition.

## Results

### Demographics

There was no significant difference between the monolinguals and bilinguals for Age in Years [*F*(1, 68) = 0.523, *p* = 0.472, *d* = 0.2, 1 – β = 0.12]^1^ and for Years of Education [*F*(1, 68) = 0.037, *p* = 0.849, *d* = 0.06, 1 – β = 0.06]. As expected, there was a significant difference in Age in Years between the young and older adults [*F*(1, 68) = 2353.2, *p* < 0.001, *d* = 11.8, 1 – β = 1.0] with a significant interaction between Age and Language Group [*F*(1, 68) = 4.776, *p* = 0.032, *d* = 0.5, 1 – β = 0.6]: Age in Years was different between young and aging older monolinguals [*F*(1, 37) = 1036.4, *p* < 0.001, *d* = 10.7, 1 – β = 1.0] and between young and aging older bilinguals [*F*(1, 31) = 1724.3, *p* < 0.001, *d* = 14.8, 1 – β = 1.0]. However, there was also a significant difference between young and older adults in Years of Education [*F*(1, 68) = 6.14, *p* = 0.016, *d* = 0.6, 1 – β = 0.71]. There was no significant interaction between Age and Language Group for Years of Education [*F*(1, 68) = 2.443, *p* = 0.123, *d* = 0.4, 1 – β = 0.36]. The difference in education between young and older adults is due to differences in years of education across generations, especially in Singapore, and was impossible to control for due to changes in the society. Hence, Years of Education was used as a covariate to address this confounding factor.

There was no significant difference on the MMSE between the monolingual and bilingual aging older adults [*F*(1, 33) = 0.113, *p* = 0.739, *d* = 0.1, 1 – β = 0.06].

Mann-Whitney tests comparing the age of acquisition for English and Tamil between the groups showed a significant difference in the age of acquisition of English between the aging older monolinguals and bilinguals (*U* = 0.000, *p* < 0.001, *r* = 0.9, 1 – β = 1.0), and the young and aging older bilinguals (*U* = 19, *p* < 0.001, *r* = 0.8, 1 – β = 1.0). There was no significant difference between the young monolinguals and bilinguals (*U* = 123.5, *p* = 0.15, *r* = 0.4, 1 – β = 0.89). As for the age of acquisition of Tamil, there was no significance difference between the young and aging older bilinguals (*U* = 141, *p* = 0.973, *r* = 0.02, 1 – β = 0.05).

The Mann-Whitney test comparing the CIMS scores did not show any significant difference between the aging older monolinguals and bilinguals (*U* = 125, *p* = 0.354, *r* = 0.17, 1 – β = 0.23).

### Background tests

Table 3 shows the results from the background tests.

**Table 3:** Untransformed mean scores *(SD)* of all participants for the background tests.

		**Monolinguals**		**Bilinguals**					
		**Young** **(*N* = 19)**	**Old** **(*N* = 20)**	**Young** **(*N* = 18)**	**Old** **(*N* = 15)**		**Main effect of Age**	**Main effect of Language Group**	**Age x Language Group interaction**
RVS	Mean *(SD)*	15.79 (3.05)	19.20 (4.20)	15.78 (2.88)	15.1 (6.43)		ns	^*^	^*^
	Min-Max	10–21	12–28	10–20	5–24				
TVL^#^	Mean *(SD)*	NA	NA	21.94 (2.91)	20.80 (5.03)		ns	NA	NA
	Min-Max	NA	NA	16–26	15–31				
ELF	Mean *(SD)*	48.53 (10.93)	57.90 (19.18)	53.39 (10.23)	42.53 (17.79)	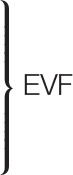	ns	^*^	^**^
	Min-Max	30–74	25–90	34–69	11–83				
ESCF	Mean *(SD)*	24.3 (3.96)	24.45 (5.48)	24.11 (4.79)	19.13 (4.72)				
	Min-Max	18–32	15–36	16–35	11–27				
TLF^#^	Mean *(SD)*	NA	NA	46.05 (10.92)	45.27 (13.18)	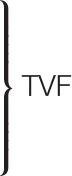	ns	NA	NA
	Min-Max	NA	NA	29–74	21–69				
TSCF^#^	Mean *(SD)*	NA	NA	17.42 (2.85)	17.07 (5.78)				
	Min-Max	NA	NA	12–24	8–28				
SA	Mean *(SD)*	35.59 (33.44)	64.33 (40.37)	25.86 (25.52)	79.79 (40.89)		^***^	ns	ns
	Min-Max	−23.09–106.75	9.05–188.4	−4.35–94.01	31.1–180.66				
BD	Mean *(SD)*	38.84 (7.32)	34.95 (9.89)	41.56 (6.11)	22.20 (6.27)		^***^	ns	–
	Min-Max	23–50	16–50	29–49	11–32				
DS	Mean *(SD)*	15.32 (2.89)	16.65 (2.89)	20.28 (2.7)	16.20 (3.78)		ns	^**^	^***^
	Min-Max	11–21	11–21	14–24	10–22				
NC	Mean *(SD)*	0.28 (0.1)	0.21 (0.06)	0.26 (0.07)	0.19 (0.08)		^***^	ns	ns
	Min-Max	0.17–0.61	0.12–0.37	0.18–0.45	0.09–0.36				

*RVS, Raven's Vocabulary Scale; TVL, Tamil Vocabulary List; ELF, English Letter Fluency; ESCF, English Semantic Category Fluency; Tamil Letter Fluency; TSCF, Tamil Semantic Category Fluency; SA, Stroop Arrow; BD, Block Design; DS, Digit Span; NC, Number comparison; EVF, English Verbal Fluency; TVF, Tamil Verbal Fluency; YM, Young Monolinguals; YB, Young Bilinguals; OM, Old Monolinguals; OB, Old Bilinguals*.

# *The Tamil background tasks were analyzed with N = 19 for young bilinguals*.

* *p < 0.05*,

** *p < 0.01*,

*** *p < 0.001*.

#### Lexical and semantic measures

In terms of vocabulary knowledge in English (RVS), there was a significant main effect of Language Group [*F*(1, 68) = 4.188, *p* < 0.05, *d* = 0.5, 1 – β = 0.55], but no significant main effect of Age [*F*(1, 68) = 1.847, *p* > 0.05, *d* = 0.3, 1 – β = 0.28]. There was a significant interaction effect between Language Group and Age [*F*(1, 68) = 4.141, *p* < 0.05, *d* = 0.5, 1 – β = 0.54]. Follow-up simple effects showed that aging older monolinguals had better vocabulary knowledge than young monolinguals [*F*(1, 68) = 6.309, *p* < 0.05, *d* = 0.6, 1 – β = 0.72] and aging older bilinguals [*F*(1, 68) = 8.026, *p* < 0.01, *d* = 0.7, 1 – β = 0.82]. There were no significant differences in the vocabulary knowledge of the young monolinguals and bilinguals [*F*(1, 68) = 0.000, *p* >0.05, *d* = 0.00, 1 – β = 0.05], and between young bilinguals and aging older bilinguals [*F*(1, 68) = 0.210, *p* > 0.05, *d* = 0.1, 1 – β = 0.074]. In terms of vocabulary knowledge in Tamil (TVL), the young bilinguals and aging older bilinguals did not differ [*F*(1, 32) = 0.696, *p* > 0.05, *d* = 0.3, 1 – β = 0.13].

The two-way ANOVA on the English Verbal Fluency test (EVF) showed a significant main effect of Language Group [*F*(1, 68) = 5.266, *p* < 0.05, *d* = 0.6, 1 – β = 0.64], but no significant main effect of Age [*F*(1, 68) = 1.852, *p* > 0.05, *d* = 0.3, 1 – β = 0.29]. There was a significant interaction effect between Language Group and Age [*F*(1, 68) = 9.208, *p* < 0.01, *d* = 0.7, 1 – β = 0.87]. Both aging older monolinguals [*F*(1, 68) = 13.685, *p* < 0.001, *d* = 0.9, 1 – β = 0.96] and young bilinguals [*F*(1, 68) = 8.886, *p* < 0.01, *d* = 0.7, 1 – β = 0.86] had better verbal fluency than aging older bilinguals. There were no significant differences between the young monolinguals and aging older monolinguals [*F*(1, 68) = 1.534, *p* > 0.05, *d* = 0.3, 1 – β = 0.24], and between the young monolinguals and young bilinguals [*F*(1, 68) = 0.284, *p* > 0.05, *d* = 0.1, 1 – β = 0.083]. The young bilinguals and aging older bilinguals did not differ in the Tamil Verbal Fluency test (TVF) [*F*(1, 32) = 0.055, *p* > 0.05, *d* = 0.09, 1 – β = 0.057].

#### Measures of cognitive abilities

A two-way ANOVA showed no significant main effect of Language Group on the Stroop Effect [*F*(1, 68) = 0.116, *p* > 0.05, *d* = 0.09, 1 – β = 0.07] and no significant interaction of Language Group and Age [*F*(1, 68) = 2.243, *p* > 0.05, *d* = 0.36, 1 – β = 0.33]. However, there was a highly significant main effect of Age on the Stroop Effect [*F*(1, 68) = 24.15, *p* < 0.001, *d* = 1.2, 1 – β = 0.999] indicating that young adults had better inhibitory abilities than aging older adults.

The Kruskal-Wallis test showed a highly significant effect of Age on the Block Design [*H*(1) = 17.985, *p* < 0.001]. There was no significant effect of Language Group [*H*(1) = 1.968, *p* > 0.05]. Follow-up Mann-Whitney tests indicated that the young bilinguals had higher scores on the Block Design than the aging older bilinguals (*U* = 2.0, *p* < 0.001, *d* = 2.1). There was no difference between the young and aging older monolinguals (*U* = 148.5, *p* > 0.025, *d* = 0.38). (A Bonferroni correction was applied, and all effects are reported at a 0.025 level of significance).

There was a significant main effect of Language Group on the Digit Span [*F*(1, 68) = 9.731, *p* < 0.01, *d* = 0.76, 1 – β = 0.89], but no significant main effect of Age [*F*(1, 68) = 3.598, *p* > 0.05, *d* = 0.49, 1 – β = 0.48]. There was a significant interaction effect between Language Group and Age [*F*(1, 68) = 14.001, *p* < 0.001, *d* = 0.91, 1 – β = 0.97]. Follow-up simple effects analyses showed the young bilinguals had a significantly better verbal short-term memory and working memory than young monolinguals [*F*(1, 68) = 24.461, *p* < 0.001, *d* = 1.2, 1 – β = 0.999], and aging older bilinguals [*F*(1, 68) = 14.623, *p* < 0.001, *d* = 0.93, 1 – β = 0.97]. There were no differences between young monolinguals and aging older monolinguals [*F*(1, 68) = 1.864, *p* > 0.05, *d* = 0.33, 1 – β = 0.29], and between aging older monolinguals and bilinguals [*F*(1, 68) = 0.187, *p* > 0.05, *d* = 0.11, 1 – β = 0.08].

There was no significant main effect of Language Group [*F*(1, 68) = 2.173, *p* > 0.05, *d* = 0.36, 1 – β = 0.32] on the Number Comparison and no significant interaction effect between Language Group and Age [*F*(1, 68) = 0.878, *p* > 0.05, *d* = 0.23, 1 – β = 0.16]. However, there was a highly significant main effect of Age [*F*(1, 68) = 25.206, *p* < 0.001, *d* = 1.2, 1 – β = 0.999], indicating that the young adults had better processing speed than the older adults.

### Pragmatic tasks

#### EPrag accuracy scores and TTRs

**Figure 1:**
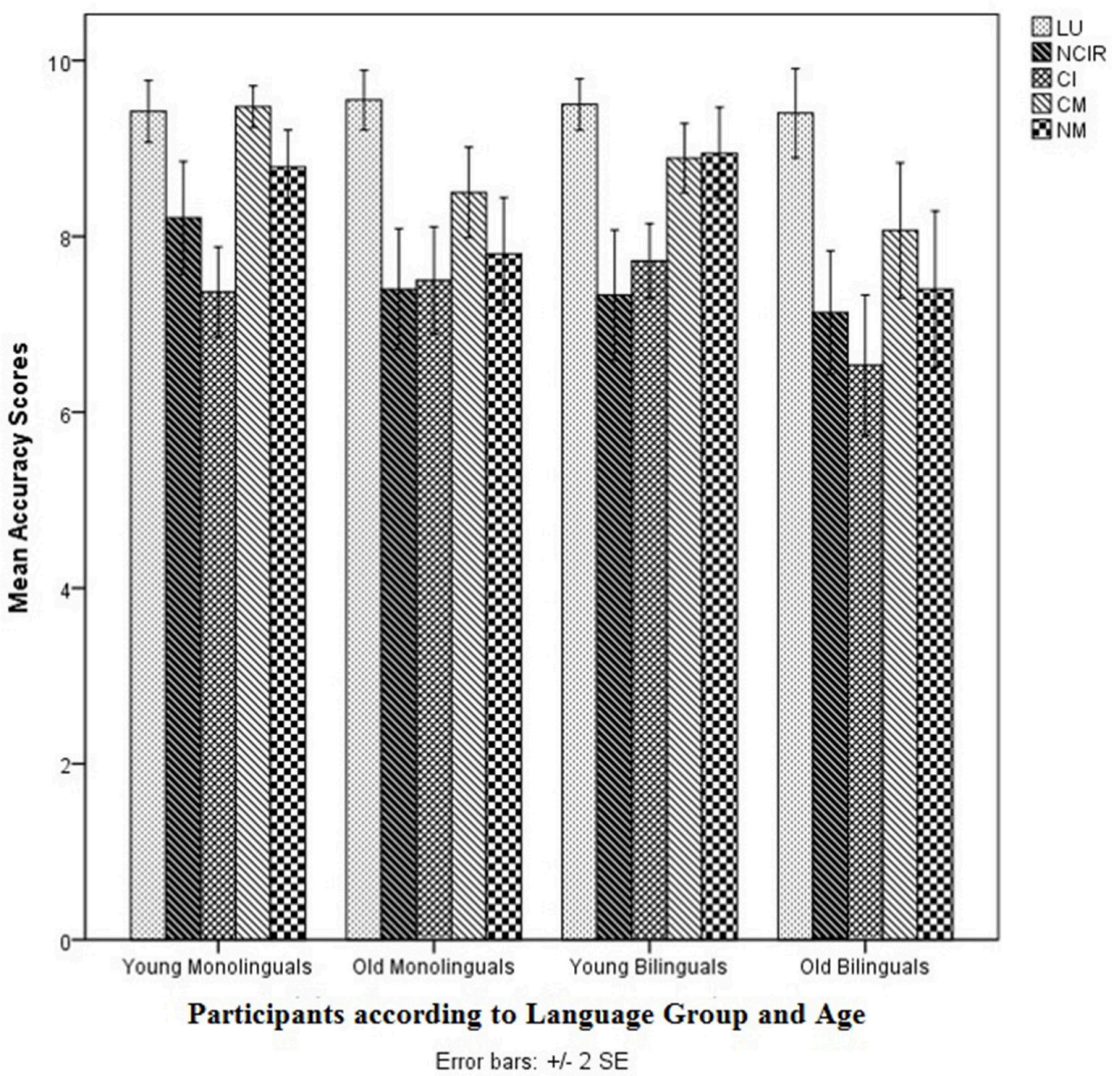
Mean accuracy scores of all participants (*n* = 72) in the EPrag task. LU, Literal Utterances; NCIR, Non-conventional Indirect Requests; CI, Conversational Implicatures; CM, Conventional Metaphors; NM, Novel Metaphors.

Figure 1 shows the participants' accuracy scores for the English Pragmatic (EPrag) task.

The MANCOVA on the accuracy scores showed a significant effect of Age on the combined dependent variables (non-conventional indirect requests, conversational implicatures, conventional metaphors, novel metaphors and literal utterances) [λ = 0.779, *F*(5, 57) = 3.225, *p* < 0.05, *d* = 1.1], indicating differences between young and aging older participants. There was no significant effect of Language Group on the combined dependent variables [λ = 0.948, *F*(5, 57) = 0.626, *p* > 0.05, *d* = 0.5], indicating that monolinguals and bilinguals performed alike, and no significant interaction effect between Language Group and Age [λ = 0.935, *F*(5, 57) = 0.793, *p* > 0.05, *d* = 0.5], indicating that monolinguals and bilinguals show the same pattern of performance. The planned comparisons for each non-literal condition separately showed that young monolinguals were significantly better than aging older monolinguals at conventional metaphors [*F*(1, 31) = 9.06, *p* = 0.005, *d* = 1.1, 1 – β = 0.9]. There was no significant difference between young bilinguals and aging older bilinguals for conventional metaphors [*F*(1, 24) = 2.072, *p* > 0.05, *d* = 0.6, 1 – β = 0.37].

**Figure 2:**
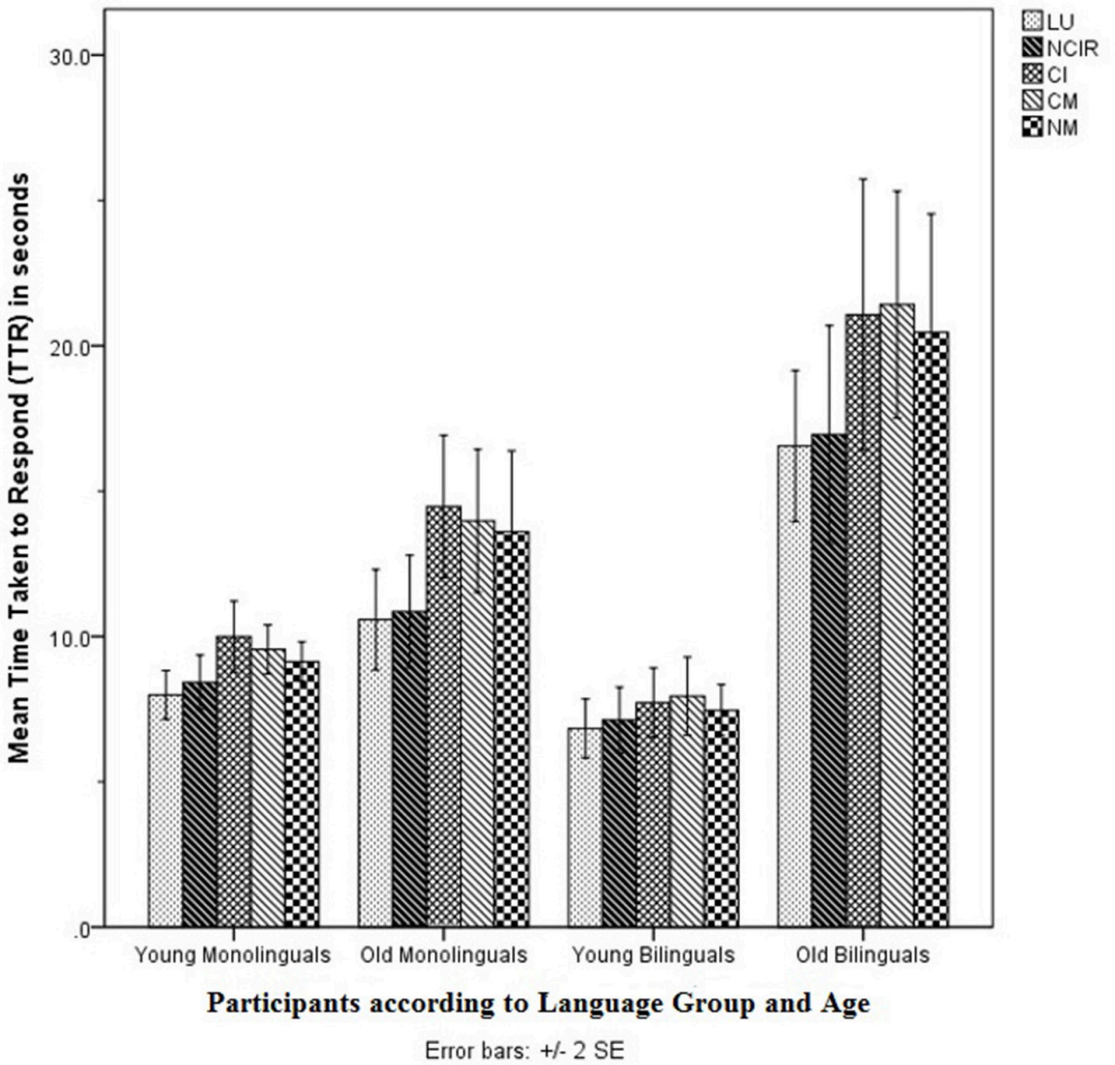
Mean time taken to respond (TTR) of all participants (*n* = 72) in the EPrag task. LU, Literal Utterances; NCIR, Non-conventional Indirect Requests; CI, Conversational Implicatures; CM, Conventional Metaphors; NM, Novel Metaphors.

Figure 2 shows the participants' TTRs for the English Pragmatic (EPrag) task.

The MANCOVA on the TTRs showed a significant main effect of Age on the combined TTRs for the non-conventional indirect requests, conversational implicatures, conventional metaphors, novel metaphors and literal utterances [λ = 0.746, *F*(5, 56) = 3.818, *p* < 0.01, *d* = 1.2], indicating differences between young and aging older participants. There was no significant main effect of Language Group on the combined TTRs [λ = 0.911, *F*(5, 56) = 1.096, *p* > 0.05, *d* = 0.6], indicating that monolinguals and bilinguals performed alike. There was no significant interaction effect between Language Group and Age [λ = 0.963, *F*(5, 56) = 0.435, *p* > 0.05, *d* = 0.4], indicating that monolinguals and bilinguals showed the same pattern of performance. The planned comparisons for each non-literal condition separately showed that young monolinguals were significantly faster than aging older monolinguals in inferring conventional metaphors [*F*(1, 30) = 7.074, *p* = 0.012, *d* = 1.0, 1 – β = 0.84], whilst there was no significant difference between the young and aging older bilinguals [*F*(1, 23) = 2.034, *p* > 0.05, *d* = 0.6, 1 – β = 0.37]. (A Bonferroni correction was applied, and the effects are reported at a 0.0125 level of significance). There were no significant differences between the young monolinguals and aging older monolinguals for the literal utterances TTR [*F*(1, 30) = 1.401, *p* > 0.05, *d* = 0.4, 1 – β = 0.26], conversational implicatures TTR [*F*(1, 30) = 5.112, *p* > 0.05, *d* = 0.8, 1 – β = 0.7] and novel metaphors TTR [*F*(1, 30) = 6.195, *p* > 0.01, *d* = 0.9, 1 – β = 0.78]. Likewise, there were no significant differences between the young bilinguals and aging older bilinguals for literal utterances TTR [*F*(1, 23) = 2.873, *p* > 0.05, *d* = 0.7, 1 – β = 0.49], conversational implicatures TTR [*F*(1, 23) = 0.716, *p* > 0.05, *d* = 0.4, 1 – β = 0.16], and novel metaphors TTR [*F*(1, 23) = 3.634, *p* > 0.05, *d* = 0.8, 1 – β = 0.59]. Planned comparison was not done for non-conventional indirect requests TTR because the independent one-way ANCOVA did not show a significant main effect of Age [*F*(1, 60) = 4.755, *p* > 0.01, *d* = 0.6, 1 – β = 0.65].

#### TPrag task accuracy scores and TTRs

**Figure 3:**
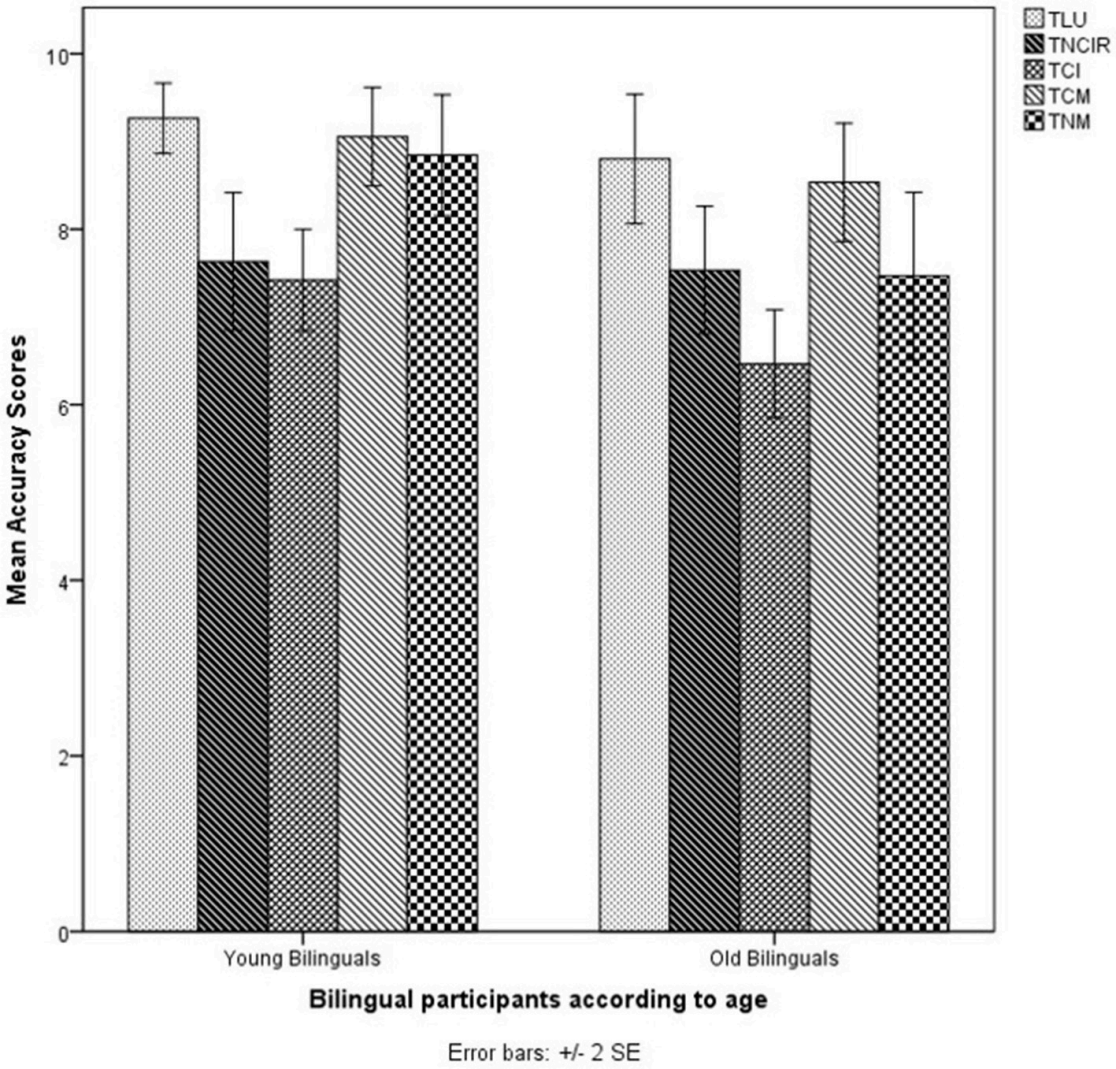
Mean accuracy scores of all bilingual participants (*n* = 34) in the TPrag task. TLU, Tamil Literal Utterances; TNCIR, Tamil Non-conventional Indirect Requests; TCI, Tamil Conversational Implicatures; TCM, Tamil Conventional Metaphors; TNM, Tamil Novel Metaphors.

**Figure 4:**
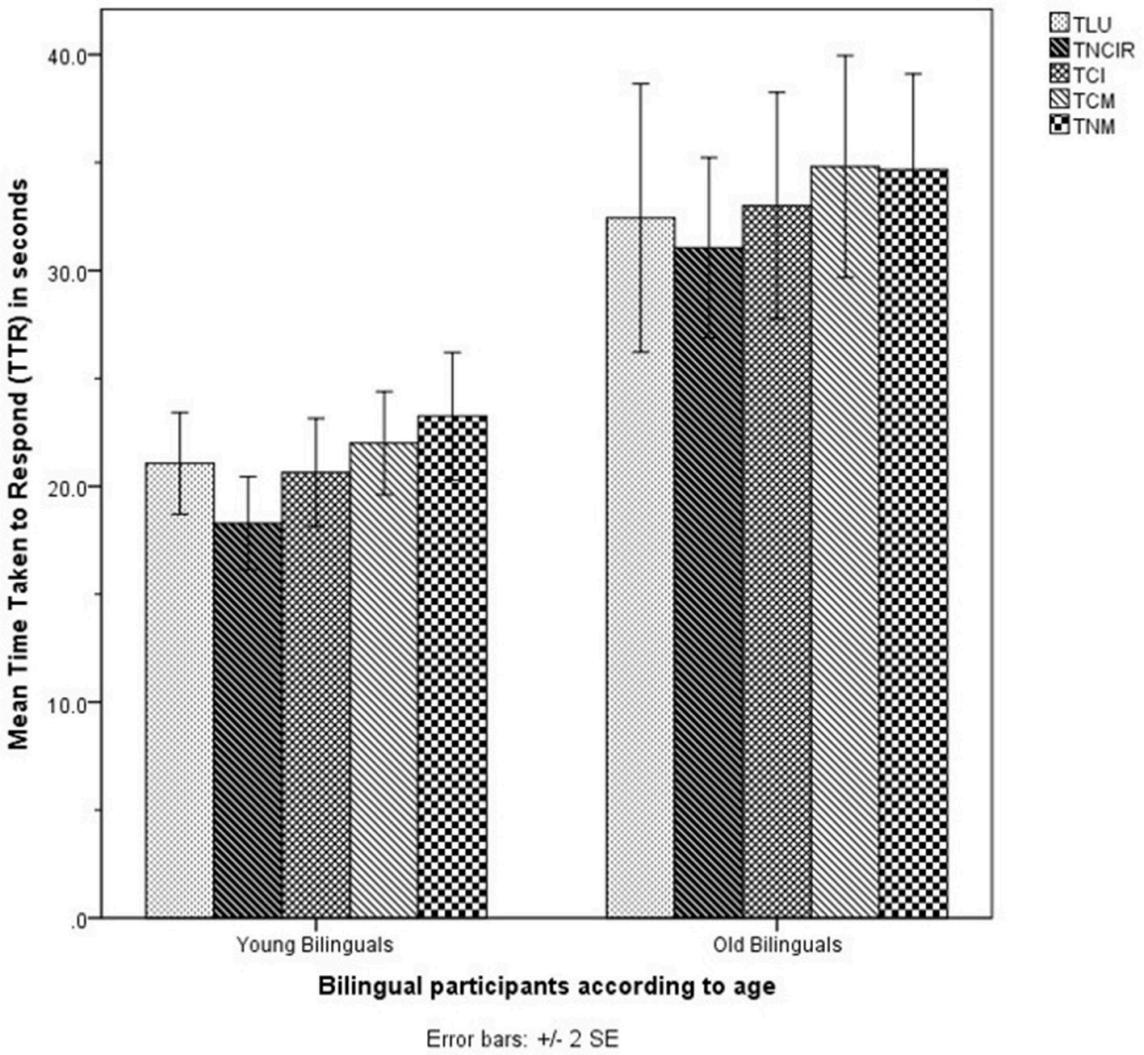
Mean time taken to respond (TTR) of all bilingual participants (*n* = 34) in the TPrag task. TLU, Tamil Literal Utterances; TNCIR, Tamil Non-conventional Indirect Requests; TCI, Tamil Conversational Implicatures; TCM, Tamil Conventional Metaphors; TNM, Tamil Novel Metaphors.

Figures 3 and 4 show the accuracy scores and TTRs for the TPrag task.

The MANCOVA on the accuracy scores showed no significant main effect of Age on the combined accuracy scores [λ = 0.873, *F*(5, 21) = 0.609, *p* > 0.05, *d* = 0.8]. Likewise, the MANCOVA on the TTRs did not show a significant main effect of Age on the combined TTRs [λ = 0.635, *F*(5, 20) = 2.3, *p* > 0.05, *d* = 1.5].

## Discussion

Everyday communication comprises of an extensive use of non-literal language, such as idioms, proverbs, metaphors, indirect requests, and conversational implicatures. Although the developed world is facing a rapidly aging population, research on the comprehension of non-literal language in aging older adults is limited and is based mainly on monolingual speakers. Whilst some studies found that aging older adults are able to access the non-literal meanings of metaphors (Ulatowska et al., 1998; Newsome and Glucksberg, 2002; Qualls and Harris, 2003; Morrone et al., 2010) and suggested that aging older adults are “as efficient” as younger adults when processing metaphors (Newsome and Glucksberg, 2002), some other studies demonstrated an age-related decline in non-literal language comprehension (Nippold et al., 1997; Uekermann et al., 2008). The differences in the findings of these studies could be related to the differences in the methodologies used, the variability in the participant populations, and the designs of the studies. Importantly, although context plays a key role in the comprehension of non-literal language, previous studies reviewed either did not present non-literal utterances within a situational context or presented them in texts that required connective inferences.

The current study aimed to fill the gap in the literature of aging older adults' pragmatic inferential abilities using non-literal utterances embedded in situational contexts. It also sought to investigate if there was a bilingual advantage in pragmatic inference-making. Young and older monolinguals and bilinguals underwent a battery of background tests to measure their vocabulary knowledge, non-verbal IQ, verbal fluency, inhibition, verbal short-term memory and working memory, and processing speed as well as completed a language use and history questionnaire to provide information such as education, age of acquisition of English and language usage. To address their pragmatic inferential abilities, participants completed an English pragmatic task that had the target literal and non-literal utterances presented in context-based vignettes that were culturally neutral. The bilinguals were, in addition, tested with a Tamil pragmatic task. Participants were tested for both accuracy and response time. After controlling for education, vocabulary knowledge, non-verbal IQ, verbal fluency, inhibition, verbal short-term memory and working memory, age of acquisition of English and processing speed, a clear effect of age on the comprehension of English conventional metaphors emerged. Planned comparisons showed that aging older monolinguals were less accurate and slower than young monolinguals on the comprehension of English conventional metaphors. Aging older bilinguals, on the other hand, were as accurate and efficient as young bilinguals on the comprehension of English conventional metaphors. Moreover, although there was no effect of Language Group (i.e., bilingualism) for any of the non-literal language types tested, this effect of age found for the monolinguals was not found for the bilinguals for any of the non-literal language types tested in the study, be it in English or Tamil.

### Understanding non-literal language as we age

In the present study, we found an age-related decline in conventional metaphor comprehension, but only for the monolinguals. Not only were the aging older monolinguals less accurate than the young monolinguals in comprehending conventional metaphors, they were also much slower when processing conventional metaphors. Past literature supports the present findings that monolingual aging older adults experience an age-related decline in non-literal language comprehension (Nippold et al., 1997; Uekermann et al., 2008). It is worth noting here that the conventional metaphors were selected based on the metaphor familiarity rating list completed by a sample of both monolingual and bilingual aging older adults, but not by the younger groups. Hence, older participants would have been guaranteed familiar with the conventional metaphors, more so than the young participants. In spite of this advantage, the aging older monolinguals were significantly less accurate and slower in inferring the metaphorical meaning of the utterances.

On the other hand, the aging older bilinguals were as accurate as the young bilinguals in terms of understanding English and Tamil metaphors (as well as the other non-literal language types tested); this is in line with studies showing that aging older adults are able to access the non-literal meanings of metaphors (Ulatowska et al., 1998; Newsome and Glucksberg, 2002; Qualls and Harris, 2003; Morrone et al., 2010). In addition, the aging older bilinguals were not significantly slower than the young bilinguals at arriving at the correct meaning of the English and Tamil metaphors. These findings suggest that aging older adults are “as efficient” as young adults when processing metaphors (Newsome and Glucksberg, 2002).

We now know that pragmatic inference-making does slow down with aging, even with processing speed attrition, cognition and other factors having been taken into account, but not for all non-literal language types and not for bilinguals.

### Bilinguals and pragmatic inference-making

The present study did not find any significant differences between the monolinguals and bilinguals in terms of pragmatic inference-making. Of the very few studies that investigated the pragmatic inference-making abilities of bilinguals, one found no bilingualism effect on conversational implicatures for L2 learners and native speakers of English (Manowong, 2011), while another found a slightly higher correlation between linguistic comprehension and pragmatic comprehension of both indirect requests and conversational implicatures for L2 learners of English with higher English language proficiency than L2 learners with lower English language proficiency (Garcia, 2004).

In the present study, the bilinguals used the English language on a daily basis and had self-assessed their English language proficiency in speaking and listening as being between “Good” to “Native-like.” The bilinguals in the present study were not disadvantaged by their “non-native speaker” status unlike the L2 leaners of English in Garcia's (2004) study and did not display a significant disadvantage in discourse processing as seen by their performance in both the literal and non-literal language types tested in the pragmatic tasks.

Although there was no overall significant effect of bilingualism on pragmatic inference-making, the findings of the present study point to a bilingual advantage when it comes to comprehending English conventional metaphors; aging older bilinguals' conventional metaphor processing was not affected by age unlike the aging older monolinguals'. As established earlier, pragmatic inferences require higher order cognitive skills (Champagne-Lavau and Joanette, 2009), and a number of studies have shown bilingualism attenuating cognitive decline associated with aging (Luk et al., 2011) and bilinguals possessing superior cognitive abilities than monolinguals even as they get older (Bialystok et al., 2006). Thus, it should come as no surprise that aging older bilinguals were not affected by age whilst processing conventional metaphors unlike their monolingual counterparts.

The sample size of the present study was small, which is one of the limitations of the study. A second limitation is that the study focused only on comprehension and did not measure the participants' production of non-literal language. Future research can compare the comprehension with the production of non-literal language by a larger sample of aging older adults and examine the effects of Language Group. This would provide a complete picture of both comprehension and production of non-literal language.

## Conclusion

The present study examined the effects of age(ing) and the effects of bilingualism on pragmatic inferences by monolingual and bilingual young and older adults. The present study has controlled for a large number of variables that can affect pragmatic inference-making. These variables include the participants' vocabulary knowledge, non-verbal IQ, education, socioeconomic status, age of acquisition of English, inhibition, verbal short-term memory and working memory, verbal fluency, and processing speed. On top of this, the young and aging older bilinguals were tested in both their languages, English and Tamil. Regardless of language, aging older bilinguals were not affected by age whilst processing literal and non-literal language. This is in direct contrast to aging older monolinguals who displayed an age-related disadvantage when confronted with conventional metaphors. This suggests a bilingual advantage in pragmatic inferences of conventional metaphors.

## Ethics statement

This study was reviewed by the School of Psychology and Clinical Language Sciences' Ethics Committee and the University Research Ethics Committee (University of Reading) and was given a favorable ethical opinion for conduct.

## Datasets are available on request

The raw data supporting the conclusions of this manuscript will be made available by the authors, without undue reservation, to any qualified researcher.

## Author contributions

SS conceived and designed the study together with TM and AB. SS created the pragmatic tasks, collected, analysed, and interpreted the data. SS together with TM and AB wrote the paper.

### Conflict of interest statement

The authors declare that the research was conducted in the absence of any commercial or financial relationships that could be construed as a potential conflict of interest.

## Footnotes

^1^Effect size and power for all analyses were calculated using G^*^Power (Version 3.1.9.2) and Lenhard and Lenhard (2016) (https://www.psychometrica.de/effect_size).
