# Supplementary material for: Comprehending Non-literal Language: Effects of Aging and Bilingualism
Source: Front Psychol. 2018 Nov 22;9:2230. doi: 10.3389/fpsyg.2018.02230 (PMC6262781; doi:10.3389/fpsyg.2018.02230)
Supplement: Supplementary file 1 [file Data_Sheet_1.PDF]

Table 1 Story board and task questions of the English Pragmatic (EPrag) task

Situational context					
**Setting**	**Background**	**Dialogue**	**Target utterance**	**Question** (shown on screen)	**Options** (shown on screen)
**Literal Utterance**					
At a job interview	A young man is attending an interview for a job. He has just entered the interview room to meet the interviewers.	Interviewee: Good morning.<br>Interviewer: Good morning.	Please sit down on that chair.	What will the young man say or do next?	1) He will say that he had been waiting for long. [possible, but wrong reaction]<br>2) He will ask the interviewers why they are there. [wrong answer]<br>3) He will sit down on the chair. [literal meaning]<br>4) He will take the chair out of the room. [wrong answer]
At a zebra crossing	A father and his young daughter are waiting to cross at the zebra crossing.	Daughter: Can we cross now?<br>Father: No. We have to wait for the cars to completely stop. (pause) They have stopped.	We can cross the road now.	What will the daughter say or do next?	1) She will hug her father and cry. [wrong answer]<br>2) She will hold her father's hand and cross the road. [literal meaning]<br>3) She will say that zebras are white with black stripes. [wrong answer]<br>4) She will ask why they are crossing the road. [possible, but wrong reaction]
At the beach	A young couple is at the beach on a sunny day.	Man: It's really very sunny today.<br>Woman: Yes, it is. Please	apply some sunblock lotion on my back for me.	What will the man say or do next?	1) He will apply the sunblock lotion on her back. [literal meaning]<br>2) He will buy some vanilla ice cream. [wrong answer]<br>3) He will say that the sun's rays are harmful to human skin. [possible, but wrong reaction]<br>4) He will open the umbrella for her. [wrong answer]
In a computer lab	Two people are in a computer laboratory working on the computer. One of them is sending an	Man: Shall I put a comma after this word?<br>Woman: Yes, and don't forget the full-stop.		What will the man say or do next?	1) He will ask her if she would like some lunch. [possible, but wrong reaction]<br>2) He will put a stamp on the letter. [wrong answer]<br>3) He will read the email out for all to hear. [wrong

Situational context					
**Setting**	**Background**	**Dialogue**	**Target utterance**	**Question** (shown on screen)	**Options** (shown on screen)
	email to their co-workers in the department.	Man: Anything else I need to correct before emailing this letter?<br>Woman: No, it's perfect now.	Click the send button to email the letter.		answer]<br>4) He will email the letter to his co-workers. [literal meaning]
In a flat	A young woman is viewing a flat to rent. The estate agent is showing her around the flat.	Estate agent: This is a big flat.<br>Woman: Yes, it seems like it. What about the furniture?<br>Estate agent: Most of the furniture stay.	Do you have your own furniture?	What will the young woman say or do next?	1) She will say she has some furniture of her own. [literal meaning]<br>2) She will say, "Do you want to buy them from me?" [wrong answer]<br>3) She will say, "I like the furniture in this flat." [possible, but wrong reaction]<br>4) She will say that she wants to look at the bathroom. [wrong answer]
At a bank	A young man is at a bank in High Street to deposit some money into his account. He has filled up the relevant form and has his money and bankbook in his hand.	Banker: Good morning.<br>Man: Good morning.	I want to deposit this money into my bank account.	What will the bank officer say or do next?	1) She will say, "Thank you. I can go shopping now." [wrong answer]<br>2) She will say, "Sure, let me withdraw the amount from your account." [wrong answer]<br>3) She will say, "Would you like to open another account with us?" [possible, but wrong reaction]<br>4) She will say, "Sure, let me update your bankbook to show the deposit." [literal meaning]
In the living room	A father and daughter are watching TV in the living room.	Father: This show is boring. Shall we switch the channel?<br>Daughter: Yes,	let's switch to Channel 5.	What will the father say or do next?	1) He will switch to Channel 8. [possible, but wrong reaction]<br>2) He will say that it's time she went to bed. [wrong answer]<br>3) He will switch to Channel 5. [literal meaning]<br>4) He will say it's time they switched off the TV. [wrong answer]

Situational context					
**Setting**	**Background**	**Dialogue**	**Target utterance**	**Question** (shown on screen)	**Options** (shown on screen)
In a photo studio	An elderly lady is at a photo studio to have her photo taken.	Photographer: So what can I do for you?<br>Lady:	I want a passport-sized photograph taken of me.	What will the photographer say or do next?	1) He will ask her to model in front of the camera. [wrong answer]<br>2) He will take a passport-sized photograph of her with his camera. [literal meaning]<br>3) He will ask her for the roll of film to develop. [wrong answer]<br>4) He will ask her to put on some lipstick. [possible, but wrong reaction]
In a courtroom	A former Member of Parliament has been charged for corruption. The prosecutor is questioning the key witness for the prosecution.	Prosecutor:	Did you see the former Member of Parliament accepting the bribe?	What will the witness say or do next?	1) She will say that she forgot to bring her spectacles to the courtroom. [wrong answer]<br>2) She will say that she has never accepted any bribe. [wrong answer]<br>3) She will say that she promises to speak the truth, the whole truth, and nothing but the truth. [possible, but wrong reaction]<br>4) She will say that she did see the former Member of Parliament accepting the bribe. [literal meaning]
In a paper factory	It is the factory policy that all machines are switched off during lunchtime. However, one machine is still operating.	Supervisor: It's lunchtime now.	Switch off the machine.	What will the worker say or do next?	1) She will switch off the machine. [literal meaning]<br>2) She will eat her lunch. [possible, but wrong reaction]<br>3) She will say that she is on a strike. [wrong answer]<br>4) She will say that too many trees are being killed to make paper. [wrong answer]
**Non-conventional Indirect Request**					
At a party	Jill is at a party.	Guy: Do you want some chips?<br>Jill: No, thank you, but	I am feeling thirsty.	What will the guy in the story say or do	1) He will get her some chips. [wrong answer]<br>2) He will ask her to dance with him. [possible, but wrong reaction]<br>3) He will get her a drink. [inferred meaning]

Situational context					
**Setting**	**Background**	**Dialogue**	**Target utterance**	**Question** (shown on screen)	**Options** (shown on screen)
				next?	4) He will say that she needs to drink something to quench her thirst. [literal meaning]
In the dining room	A husband and wife are in the dining room. The room is rather warm.	Wife: It's way	too warm in this room.	What will the husband say or do next?	1) The husband will say, "Yes, it is awfully warm." [literal]<br>2) The husband will open the windows so that cool air can enter the room. [inferred meaning]<br>3) The husband will have a shower. [wrong answer]<br>4) The husband will check the wife's temperature. [possible, but wrong reaction]
At the clinic	Jane is at the clinic. She is visiting her doctor.	Patient: I've had this pain in my tummy for 2 days now.<br>Doctor: Well, I'll need to have a look at that.	The sheet on the examination table has just been changed.	What will the patient say or do next?	1) She will check the sheet on the examination table to see if it has been changed. [literal]<br>2) She will say, "The examination table sheets must be cheap, if you can change them so often." [possible, but wrong reaction]<br>3) She will walk out of the room. [wrong answer]<br>4) She will lie down on the examination table. [inferred meaning]
At the supermarket	Sally is at the supermarket to buy milk of brand X.	Sally (to sales assistant): Hi.	Do you sell brand X?	What will the sales assistant say or do next?	1) He will tell her in which aisle in the supermarket brand X is in. [inferred meaning]<br>2) He will say, "Yes, we do sell brand X." [literal meaning]<br>3) He will say, "Is brand X low-fat milk?" [possible, but wrong reaction]<br>4) He will tell her that he hates working at the supermarket. [wrong answer]
At the car park	Jack is walking towards his car at the car park where he sees Diane in her car looking helpless.	Jack: Hello, Diane. Is there a problem?<br>Diane: Hello, Jack. I can't start my car. I think the		What will Jack say or do next?	1) He will offer to take her home in his car. [inferred meaning]<br>2) He will say, "No, I am not in a hurry to get somewhere. Why do you ask?" [literal meaning]

Situational context					
Setting	Background	Dialogue	Target utterance	Question (shown on screen)	Options (shown on screen)
		battery is flat. I need to get back home; I am expecting a parcel delivery in ½ an hour.<br>Jack: Oh dear!<br>Diane:	Are you in a hurry to get somewhere?		3) He will get into his car and drive off. [wrong answer]<br>4) He will examine her car tyres. [possible, wrong reaction]
At the pharmacy	A man is at the pharmacy to buy medication for his cold.	Sales girl: What can I do for you?<br>Man:	I have a cold.	What will the pharmacist say or do next?	1) She will say, “Yes, you do sound like you have a cold.” [literal meaning]<br>2) She will recommend some medication for the cold. [inferred meaning]<br>3) She will give him a piece of tissue. [possible, but wrong reaction]<br>4) She will ask him why he is at the pharmacy. [wrong answer]
On a bus	Lisa has just boarded a bus. She places her bag on the empty seat next to her. A few minutes later, a man boards the bus and looks for a seat.	Man: Hi.	Is anyone sitting here?	What will Lisa say or do next?	1) She will say her bag is on that seat. [possible, but wrong reaction]<br>2) She will say, “No, no one is sitting here.” [literal meaning]<br>3) She will ask him if he has paid the bus fare. [wrong answer]<br>4) She will remove the bag and say, “No, please have the seat.” [inferred meaning]
In a primary school’s general office.	Some parents and their children are waiting to see the school principal. A father and his young son are first in the line. While waiting, the little boy needs to go to the toilet and so the	Father (to receptionist): I’m taking my son to the toilet that is just round the corner;	he needs to use the toilet.	What will the receptionis t say or do next?	1) She will say, “Yes, please take him to the toilet if he needs to urinate/pee.” [literal meaning]<br>2) She will say, “Don’t forget to flush!” [possible, but wrong reaction]<br>3) She will say, “The canteen is still open, if you want a drink.” [wrong answer]<br>4) She will say, “Go ahead, you won’t lose your

Situational context					
**Setting**	**Background**	**Dialogue**	**Target utterance**	**Question** (shown on screen)	**Options** (shown on screen)
	father approaches the receptionist…				place in the line; you will still be the first to see the principal.” [inferred meaning]
Taking a taxi.	A woman has just got into a taxi. She wants to go to No. 8 High Street.	Woman:	Do you know where No. 8 High Street is?	What will the taxi driver say or do next?	1) He will say, “Yes, I know where it is.” [literal meaning]<br>2) He will park his taxi and get out. [wrong answer]<br>3) He will drive her to No. 8 High Street. [inferred meaning]<br>4) He will say, “Please put on your seat belt.” [possible, but wrong reaction]
In a small restaurant	Susan and her friend are in a restaurant having their dinner when Susan drops her fork. She calls the waiter over.	Susan: Hi.	I just dropped my fork.	What will the waiter say or do next?	1) He will get her another fork. [Inferred meaning]<br>2) He will say, “Do you want a spoon?” [possible, but wrong reaction]<br>3) He will look at the floor and say, “Yes, the fork is on the floor.” [literal meaning]<br>4) He will get her something to eat. [wrong answer]
**Conversational Implicature**					
At the playground	Grandpa is at the playground with his grand-daughter. She is afraid to get on the swing.	Granddaughter: I am so scared to get on the swing. What if I were to fall?<br>Grandpa: Don’t be scared. You won’t fall. Now,	we can’t be standing next to the swing the whole day.	What will the grand-daughter do or say now?	1) She will give grandpa a hug. [possible, but wrong reaction]<br>2) She will say, “Okay, grandpa, you help me get onto the swing.” [inferred meaning]<br>3) She will say, “Let’s have some ice-cream!” [wrong answer]<br>4) She will say, “No, we can’t stand next to the swing the whole day as that would be very tiring.” [literal meaning]
In a dental clinic	John is late for his appointment. He tries hard to convince the dental nurse to let him see the dentist as	Nurse: You will have to wait till the other patients have been seen. If someone cancels or comes in much		What will the nurse say or do next?	1) She will say, “Oh, a strike, how wonderful!” [wrong answer]<br>2) She will say, “Oh, not your fault for being late then; I’ll slot you in somehow.” [inferred meaning]

Situational context					
**Setting**	**Background**	**Dialogue**	**Target utterance**	**Question** (shown on screen)	**Options** (shown on screen)
	soon as possible even though he has missed his appointment.	later, you can have that appointment slot. John: I'm sorry, but	the bus drivers were on a strike.		3) She will say, "So the bus drivers didn't drive the buses today." [literal meaning] 4) She will say, "Strikes are so inconvenient sometimes." [possible, wrong reaction]
At a hospital	Vicky is at the hospital. She hurt herself coming down the stairs, and now is at the hospital for an X-ray to be taken of her ankle.	Radiologist: Please go to the changing room and change out of your clothes into this x-ray gown. Vicky: I am here	just for an ankle x-ray.	What will the radiologist say or do next?	1) He will say, "The gown is worn with the opening at the back." [possible, but wrong reaction] 2) He will say, "You will need to remove only your jeans and put on the x-ray gown" [inferred meaning] 3) He will say, "Yes, we will take an x-ray of your ankle." [literal meaning] 4) He will say, "We could take an x-ray of your chest too." [wrong answer]
At the bus-stop.	Two friends are waiting for the bus. It then starts to rain.	Boy: Oh nooo. It's raining. Girl: Well,	I am glad that this bus-stop is sheltered.	What will the boy say or do next?	1) The boy will go out into the rain. [wrong answer] 2) The boy will look up and say, "Yes, you are right; this bus-stop has a shelter." [literal meaning] 3) The boy will say, "Yes, I, too, am glad I am not going to get wet in the rain." [inferred meaning] 4) The boy will board the bus. [possible, but wrong reaction]
At the cinema	Two people coming out of the cinema after watching a movie.	Man:	Why do I feel like I just wasted my money?	What will the woman say or do next?	1) She will say, "Tell me about it. What a boring movie!" [inferred meaning] 2) She will give him some money. [wrong answer] 3) She will say, "That's because you just spent money on a movie and not put it into a bank." [literal meaning] 4) She will try to cheer him up. [possible, wrong reaction]
At the canteen	Tony is buying his lunch at the office canteen.	Tony: Do you have fish? Canteen lady:	It has been raining	What will Tony say	1) He will say, "Yes, it has been very wet for weeks now." [literal meaning]

Situational context					
**Setting**	**Background**	**Dialogue**	**Target utterance**	**Question** (shown on screen)	**Options** (shown on screen)
			non-stop for weeks now.	or do next?	2) He will say, "Why didn't you take the week off?" [wrong answer]<br>3) He will say, "No fish eh. So what do you have?" [inferred meaning]<br>4) He will say, "I eat only fish." [possible, but wrong reaction]
In the library	Two students are in the school library. They have one hour to work on their project, but they are talking and giggling. Other library users are disturbed by this. The librarian approaches them and says	Librarian:	This is not a playground.	What will the students say or do next?	1) They will say, "Okay, we will go to the playground." [possible, wrong reaction]<br>2) They will say," Sorry, we will keep quiet and not make any noise." [inferred meaning]<br>3) They will say, "No, this place is full of books; it's not a playground." [literal meaning]<br>4) They will say, "The library needs more books." [wrong answer]
At the police station	A woman is at a police station to make a report about a stolen mobile phone.	Woman: I was at AZ shopping center this afternoon and someone stole my mobile phone from my bag.<br>Policeman: Did you see the person who took the phone out of your bag?<br>Woman: The baby was hungry and crying;	I was trying to calm the baby and feed her.	What will the policeman say or do next?	1) He will write on the police report that the baby was hungry and crying. [literal meaning]<br>2) He will advise her on how to be a better parent. [wrong answer]<br>3) He will tell her to be more careful in the future with her valuables. [possible, wrong reaction]<br>4) He will write on the police report that she did not see who took the phone out of her bag. [inferred meaning]
In a classroom	The students, one by one, are placing their completed homework on the teacher's	Teacher: Chan,	do you need a special invitation?	What will the student say or do	1) The student will say, "All the teachers are giving us too much homework." [possible, wrong reaction]<br>2) The student will say, "Yes, please give me the

Situational context					
**Setting**	**Background**	**Dialogue**	**Target utterance**	**Question** (shown on screen)	**Options** (shown on screen)
	table. One of the students, Chan, is daydreaming.			next?	invitation card." [literal meaning]<br>3) The student will say, "Is it break-time already?" [wrong answer]<br>4) The student will say, "Sorry, here is my completed homework." [inferred meaning]
At the corner shop	A woman is buying some groceries from a small corner shop in her neighborhood.	Shopkeeper: That'll be 35.90.<br>Woman: Here you go, 35.90. Next time	I'll get the tomatoes from a jewelry shop!	What will the shopkeeper say or do next?	1) The shopkeeper will say, "Indian women love jewellery!" [wrong answer]<br>2) The shopkeeper will say, "Are they selling tomatoes in the jewellery shops nowadays?" [literal meaning]<br>3) The shopkeeper will say, "There is a jewellery shop just down the road." [possible, wrong reaction]<br>4) The shopkeeper will say, "Yes, the price of tomatoes has gone up a lot." [inferred meaning]
**Conventional Metaphor**					
In a coffee shop	A man and a woman are talking about their wealthy neighbor.	Man: Did you know that our rich neighbor just got married?<br>Woman: Of course! Everyone's talking about it.<br>Man: I heard his new wife is	a gold-digger.	What will the woman say or do next?	1) She will say, "I hope he realizes that his new wife is just after his money." [inferred meaning]<br>2) She will buy a cup of coffee for her husband. [wrong answer]<br>3) She will say, "Which gold mine does she work in?" [literal meaning]<br>4) She will offer to shop for the wedding gift. [possible, but wrong reaction]
At the post office	Lucy is at the post office.	Sales assistant: Hello.<br>Lucy: I want to post this package to Japan. How much will it cost?<br>Sales assistant: Let me check. (pause). It will cost		What will the sales assistant do or say next?	1) He will say, "Well, hold on to your head to stop it." [literal meaning]<br>2) He will say, "Cost of everything has increased, including postage." [possible, but wrong reaction]<br>3) He will say, "I'll write down the rates; that would be less confusing for you." [inferred meaning]

Situational context					
**Setting**	**Background**	**Dialogue**	**Target utterance**	**Question** (shown on screen)	**Options** (shown on screen)
		you 60 for a special same-day delivery, 44 for an express 2-day delivery, 31 for a non-standard 3-day delivery and 27 for a standard 5-day delivery.<br>Customer: Oh dear. Now	my head is spinning.		4) He will say, "Would you like some panadols?" [wrong answer]
In an office pantry	Two colleagues are gossiping about their new co-worker	Male colleague: Did you meet the new guy?<br>Female colleague: Yes, I met him yesterday during lunch break.<br>Male colleague: He didn't know how to operate the photocopier.<br>Female colleague: Well, what did you expect? He is	a greenhorn.	What will the male colleague say or do next?	1) He will say, "Well, he looks a bit green, but not like a horn." [literal meaning]<br>2) He will say, "Yes, you are right; he doesn't know anything about his job." [inferred meaning]<br>3) He will say, "Have you had your lunch?" [wrong answer]<br>4) He will say, "Do you think he is cute?" [possible, but wrong reaction]
At the park.	Tom and Mary are taking a stroll at the park where they see Jane seated on a bench and in pain.	Tom/Mary: Hello, Jane.<br>Jane: Hello, Mary. Hello Tom.<br>Tom: What's the matter?<br>Jane: It's my knee.	It's killing me.	What will Tom and Mary say or do next?	1) They will offer their sympathies towards her pain. [inferred meaning]<br>2) They will offer to call the police so that the killer can be caught. [literal meaning]<br>3) They will say how a walk in the park is good for health. [possible, wrong reaction]<br>4) They will ask her if she comes to the park often. [wrong answer]
At a wedding party	Francis is at his cousin's wedding dinner. He sees an old friend and approaches her.	Francis: Hi, Susan! How are you?<br>Susan: I'm doing good. Thank you. How are you?		What will Susan say or do next?	1) She will say, "Yes, I will be singing all the high notes later this evening." [literal meaning]<br>2) She will say, "You didn't expect me here tonight, did you?" [possible, but wrong reaction]

Situational context					
**Setting**	**Background**	**Dialogue**	**Target utterance**	**Question** (shown on screen)	**Options** (shown on screen)
		Francis: I'm great. I'm glad you came today. Your presence is	the high note of the evening.		3) She will say, "The party would still be great without me." [inferred meaning]<br>4) She will say, "When will they cut the wedding cake?" [wrong answer]
In an aeroplane	All passengers have taken their seats in the aeroplane. While waiting for the flight to takeoff, two flight attendants talk about their job.	Steward: This is my 6th month as a flight attendant. So how long have you been with Penguin Airlines?<br>Stewardess: I've been with this airline for almost 7 years now.<br>Steward: You must love this job.<br>Stewardess: It pays well, but the frequent travelling is very tiring. This	job is a jail.	What will the male flight attendant say or do next?	1) He will say, "I can't wait for the plane to takeoff!" [possible, but wrong reaction]<br>2) He will say, "Does that mean that the passengers are prisoners?" [literal meaning]<br>3) He will say, "Why do we have to put on our seatbelts?" [wrong answer]<br>4) He will say, "Really? Have you considered other positions in the airlines?" [inferred meaning]
At a crime scene	A lady has been shot dead. The detective is questioning the husband, who had found her dead when he came home earlier than usual.	Detective: What time did you come back home?<br>Husband: I came home earlier than usual to check on her. She didn't answer her phone and I got worried. So I came home earlier.<br>Detective: Ummm…why did you get come home earlier than usual when she didn't answer her phone?<br>Husband: Because I got worried. Come on detective,		What will the detective say or do next?	1) She will say that his wife was shot dead because she did not cook dinner. [wrong answer]<br>2) She will say, "I did not say that you are lying." [inferred meaning]<br>3) She will say that she could give him the recipe for the story. [literal meaning]<br>4) She will say, "The telephone line has been cut." [possible, but wrong reaction]

Situational context					
**Setting**	**Background**	**Dialogue**	**Target utterance**	**Question** (shown on screen)	**Options** (shown on screen)
		I am not	cooking up a story.		
In the garden	Two friends are chatting in the garden about another friend who had killed herself.	Man: She killed herself in the bathroom; her husband was sleeping in the room.<br>Women: Well, according to my source, before he went to the room to sleep, he had	planted the idea of suicide in her mind.	What will the male friend say or do next?	1) He will say, "Well, the sneaky bastard!" [inferred meaning]<br>2) He will say, "Would you like another glass of lemonade?" [wrong answer]<br>3) He will say, "But he would have needed enough water and sunlight after planting." [literal meaning]<br>4) He will say, "How long was he asleep?" [possible, but wrong reaction]
At a nursery	Two parents are talking about their toddlers. One has just started nursery.	Parent 1: So how has your little boy's first week at the nursery been?<br>Parent 2: Well, he cried and screamed the first 3 days; it was just so horrible.<br>Parent 1: He must have missed you.<br>Parent 2: But he is much better now;	he has been an angel.	What will the other parent say or do next?	1) He will say that she is lucky that her little boy has adapted so soon to the nursery. [inferred meaning]<br>2) He will tell her to keep it a secret that her son is an angel as people might bother them for blessings. [literal meaning]<br>3) He will say that he wished he could have gone to a nursery when he was a child. [wrong answer]<br>4) He will say that childcare is indispensable, but very expensive nowadays. [possible, but wrong reaction]
In a parliament session	Two politicians were arguing in parliament. They were arguing over a population policy that needed parliament's approval.	Politician 1: We have an ageing population. We need to allow more immigrants into the country to support the elderly.<br>Politician 2: I don't disagree we have an ageing population, but you are just	barking up the wrong tree.	What will the male politician say or do next?	1) He will say that he is paid more as a politician than she is. [wrong answer]<br>2) He will say that he is not a dog to bark at trees. [literal meaning]<br>3) He will say that the new immigrants will be better educated. [possible, but wrong reaction]<br>4) He will say that if she can offer a better solution, she should. [inferred meaning]

Situational context					
**Setting**	**Background**	**Dialogue**	**Target utterance**	**Question** (shown on screen)	**Options** (shown on screen)
**Novel Metaphor**					
At a Yoga Meditation Centre	Jenny is at the Yoga Centre. She has just finished her yoga session and is packing up to rush home.	Yoga Trainer: You seem to be in a hurry.<br>Jenny: Yes, I have to get back home to cook dinner. My daughter is home and she will be hungry.<br>Yoga Trainer: She could eat whatever was left over from lunch.<br>Jenny: She would have finished the leftovers, and still be hungry.	She is a sinkhole!	What will the man say or do next?	1) He will start to meditate. [wrong answer]<br>2) He will say, "Did you know sinkholes are formed when soil beneath the surface moves into gaps formed by dissolving limestone?" [literal meaning]<br>3) He will say, "Well, growing children need more food" [inferred meaning]<br>4) He will say, "How is she doing at school? [possible, but wrong reaction]
At a birthday party	Two young people are at a friend's birthday party.	Male: This party is great!<br>Female: Yes, I just love the games.<br>Male: Hey, why isn't John here?<br>Female: Oh, he wasn't invited. This is a party with games and dancing, and	he is just drapes!	What will the boy say or do next?	1) He will say, "John must be feeling lonely." [possible, but wrong reaction]<br>2) He will walk over to the windows to look for John. [literal meaning]<br>3) He will get her a drink. [wrong answer]<br>4) He will say, "I know. He would have just sat in a corner." [inferred meaning]
At a funeral	Two friends are at the funeral of a good friend, Lisa. They are talking about the Lisa's relatives.	Female: Lisa sure had many friends and relatives.<br>Male: Plenty of relatives. She never knew she had that many nephews and nieces till she was diagnosed with cancer.<br>Female: They must love her		What will the lady say or do next?	1) She will say that Lisa must have been too weak to sweep the floor. [literal meaning]<br>2) She will start to cry. [possible, but wrong reaction]<br>3) She will say that it is a pity that some people care about money only. [inferred meaning]<br>4) She will say that Lisa must have died happy knowing that her relatives love her. [wrong answer]

Situational context					
**Setting**	**Background**	**Dialogue**	**Target utterance**	**Question** (shown on screen)	**Options** (shown on screen)
		a lot.<br>Male: Ha, Ha. When a rich aunt is dying and about to write her will,	these vacuum cleaners will show up.		
At the airport	A honeymoon couple is waiting to check-in their luggage at the airport.	Wife: You have yet to tell me which hotel we'll be staying in.<br>Husband: I've got us the honeymoon suite at the Shangri-La hotel.<br>Wife: But that's sooooo expensive. Why?<br>Husband: Because	I am so electrocuted with love.	What will the wife say or do next?	1) She will check-in their luggage. [possible, but wrong reaction]<br>2) She will say, "How did it feel to have electricity running through you?" [literal meaning]<br>3) She will say she is fortunate to marry someone who loves her so much. [inferred meaning]<br>4) She will say, "They have increased the electricity tariff again!" [wrong answer]
At the theatre	A group of friends are at the theatre to watch a musical. However, due to some technical problems, the show is delayed and the audience has yet to sit inside the theatre.	Man: This is ridiculous. We have been waiting for so long.<br>Woman: And the foyer is so overcrowded and chaotic now.	It's a kitchen with inexperienced cooks.	What will the man say or do next?	1) He will say, "Yes, let's sing and dance to entertain the audience." [wrong answer]<br>2) He will say, "Yes, there might just be a stampede." [inferred meaning]<br>3) He will say, "Yes, the restaurant over there has just hired inexperienced cooks." [literal meaning]<br>4) He will say, "Yes, I like coming to this theatre." [possible, but wrong reaction]
In the kitchen	Two foreign chefs are discussing their experience in America.	Male Chef: I love America.<br>Female Chef: Yes, I love the people in America. America is	a big pot of vegetable soup.	What will the man say or do next?	1) He will say, "Many people from so many different countries." [inferred meaning]<br>2) He will say, "Keep stirring the pot." [possible, but wrong reaction]<br>3) He will say, "So many different vegetables to

Situational context					
**Setting**	**Background**	**Dialogue**	**Target utterance**	**Question** (shown on screen)	**Options** (shown on screen)
					make one delicious soup." [literal meaning]<br>4) He will say, "Why do Americans like soup?" [wrong answer]
In the judge's chamber	A couple has filed for a divorce. Their lawyers are in the judge's chambers representing them.	Judge: Why is your client applying for divorce?<br>Lawyer: She believes that her husband does not love her and had never loved her. In fact, she has proof that he has another family. So basically, he had	flown her on an aeroplane.	What will the judge say or do next?	1) She will say, "Did he pay for the airfare?" [literal meaning]<br>2) She will say, "Does her husband admit to deceiving her?" [inferred meaning]<br>3) She will say, "Does her husband have proof of her infidelity? [wrong answer]<br>4) She will say, "Is he remorseful?" [possible, but wrong reaction]
In an examination hall	Two students who have just finished a maths examination are walking out of the examination hall.	Student 1: Question 5 was so difficult.<br>Student 2: I know. I had	to twist my head to complete that question.	What will the girl say or do next?	1) She will say, "Same here, I really worked hard at that question." [inferred meaning]<br>2) She will say, "I love to twist." [wrong answer]<br>3) She will say, "How did you manage to turn your head back the right way?" [literal meaning]<br>4) She will say, "Well, it's time I got home." [possible, but wrong reaction]
At the circus	A man is at the ticket booth buying tickets for the circus. While waiting for the transaction to be completed, he chats with the ticket sales staff.	Man: Looks like it has been a busy day for you. And there is still a long queue for the tickets.<br>Ticket sales staff: Well, I've been in this booth selling tickets for the last 8 hours without even a break. I've been	spinning in the dryer almost the whole day.	What will the man say or do next?	1) He will ask if the clown has done the laundry for the day. [wrong answer]<br>2) He will ask her how she got into the dryer. [literal meaning]<br>3) He will inform the customer behind him that the tickets are sold out. [possible, but wrong reaction]<br>4) He will say that he is sorry to hear that she has so much work. [inferred meaning]

Situational context					
**Setting**	**Background**	**Dialogue**	**Target utterance**	**Question** (shown on screen)	**Options** (shown on screen)
At the train station	The station master and his assistant have been kept busy at the train station as it is peak period. The station master shouts at a young passenger for running along the platform.	Asst: Wow. This is the first time you have yelled at a passenger.<br>Master: Yes. The silly fella could have gotten hurt or cause hurt to another. The platform is crowded today.<br>Asst: of course, silly fella could have caused a commotion. But I have never seen you	pierce your pimple before.	What will the station master say or do next?	1) He will say that acne creams don't seem to clear his pimples. [literal meaning]<br>2) He will say that it is his day off tomorrow. [possible, but wrong reaction]<br>3) He will say that nothing has made him lose his temper before. [inferred meaning]<br>4) He will say that he hates his uniform. [wrong answer]

[ ] indicates that the written text is not seen on screen or heard.
